# Incorporating Environmental Pollution and Human Development in the Energy-Growth Nexus: A Novel Long Run Investigation for Pakistan

**DOI:** 10.3390/ijerph17145154

**Published:** 2020-07-17

**Authors:** Nabila Abid, Jianzu Wu, Fayyaz Ahmad, Muhammad Umar Draz, Abbas Ali Chandio, Hui Xu

**Affiliations:** 1 School of Management, Lanzhou University, Lanzhou 730000, China; nabila2017@lzu.edu.cn (N.A.); jzwu@lzu.edu.cn (J.W.); 2 School of Economics, Lanzhou University, Lanzhou 730000, China; fayyaz@lzu.edu.cn; 3 Canadore College, Canadore at Stanford, 930 Progress Avenue, Scarborough, ON M1G 3T5, Canada; umardraz2626@gmail.com; 4 College of Economics, Sichuan Agricultural University, Chengdu 611130, China; alichandio@sicau.edu.cn

**Keywords:** energy consumption, economic growth, CO_2_ emissions, human development index

## Abstract

Energy acts as a catalyst to boost the human development index (HDI) in a country. However, the overuse of energy leads to environmental deterioration, which is a byproduct of economic development. Due to the utilization of non-renewable energy sources for a long time, worldwide environmental conditions have become alarming. This study investigates the relationship between renewable and non-renewable energy consumption, economic growth, environmental sustainability, and the human development index (HDI) in Pakistan. The investigation incorporates population growth and technology variables to form a multivariate framework. We use a fully modified ordinary least squares (FMOLS) approach to time-series data from 1990–2017. To check the robustness of estimations, we apply the Gregory–Hansen test with a causality test under the VECM to confirm this association’s directions. Our findings confirm that non-renewable energy sources have a positive association with economic growth and CO_2_ emissions. However, human development, technology, and renewable energy boost economic development and reduce environmental pollution in Pakistan. The co-integration results confirmed the long run connectivity among all variables. The causality outcomes support the bidirectional causality between renewable and non-renewable energy consumption, economic growth, and CO_2_ emissions, both in the short and long run. These outcomes suggest that Pakistan should focus on energy shifts and gradually increase the share of renewables in its energy mix under the China Pakistan Economic Corridor (CPEC). Additionally, the government should increase human and technological development to enhance economic and environmental sustainability.

## 1. Introduction

The efforts to combat environmental deterioration and conserve the environment are a challenging aspect for developing and developed nations around the world [1]. In recent years, economies have tried to balance the sustainable environment with economic growth. However, the association between economic progress and the environment is connected through the association between energy consumption and economic growth [2,3]. Energy consumption and economic growth are identified with each other, but the causality direction between two remained unclear across countries, datasets, and methodologies [4,5].

Pakistan is an emerging country that has been striving since its independence to enhance economic growth. The extreme energy crisis has caused a significant shock to Pakistan’s gross domestic product (GDP) in recent years. The rate of energy consumption has increased in the last two decades, and Pakistan has witnessed an 80% rise in energy demands in the previous 15 years [6]. Pakistan is utilizing traditional energy resources to meet its rising energy demands [7].

Non-renewable energy contributes 64% to the overall energy mix of Pakistan [8]. The coal, oil, and gas reserves in Pakistan can produce energy for decades, and utilization of these traditional resources may lower production costs and boost economic conditions by reducing dependence on imported coal [9,10], but at the same time, it increases concern for environmental sustainability [11].

Shahbaz et al. [12] stated that fossil fuel consumption is having detrimental impacts on Pakistan’s environment. In the 1997 Kyoto protocol, Pakistan was among the countries that agreed to protect the environment by slowly replacing non-renewables with renewables to minimize greenhouse gases (GHGs). In recent decades, a steady transition from traditional to cleaner energy sources has been observed globally. Pakistan has excellent renewable potential, which provides a perfect substitute for conventional energy sources, but unfortunately, this area has received little attention from authorities [13].

Renewable and non-renewable energy consumption are key drivers to achieve economic prosperity in a country [14]. Besides economic growth, energy is also needed for human well-being; the absence of energy services is an indication of poor human development in a country [15].

Economists give greater emphasis to economic growth over time, but the remaining targets of sustainable development are mainly being compromised [16]. Energy consumption increases the economic activities in a country; however, during this process, large amounts of toxic substances are released into the environment. Many economies appear to ignore the human development aspect while addressing the environmental issue. High-HDI regions spend relatively more funds and focus on environmental sustainability with reducing the fossil fuels and promoting the clean sources of energy, while poor countries with low HDI generally depend on traditional energy and spend less on modern technologies to reduce CO_2_ emissions from fossil fuels. The overall environmental, health and economic conditions of low HDI nations are far below than of the high ones. Thus, the impact of HDI on sustainable development is substantial and interesting to explore for developing nations [17].

The underlying characteristics of emerging technology also have a substantial impact on energy, so setting appropriate goals is essential for beginning the path to sustainability [18].

In previous studies, little emphasis has been given to consider human development and technology aspects with the economic, environmental, and energy perspective. HDI is a parameter that classifies a country into one of the three stages of development: developed, developing, and underdeveloped. The HDI measures the life standards and health facilities offered to individuals in a country. Human needs must be considered as a primary objective to achieve in a country, and environmental protection must remain in the second position [19]. The study chooses Pakistan as a case for carrying out this research for the following reasons.

First, Pakistan is facing a severe crisis in the energy sector, which costs 7% of the total GDP of Pakistan [20]. In 2013, China announced the world’s biggest project, the Belt and Road Initiative (BRI), under which China aimed to develop six economic corridors, and the China Pakistan Economic Corridor (CPEC) is a critical aspect of it. Out of 21 projects, six will produce electricity from non-renewable resources, which will meet 67% of Pakistan’s energy demand, and 15 are based on renewable energy sources [21]. This study adds to the literature by studying the causal correlation between energy consumption separately in the context of energy resources (renewable and non-renewable consumption), economic growth, environmental sustainability, human development index, technology, and population growth for Pakistan and by employing different sets of econometric methodologies.

Second, Pakistan formulated its first environmental protection policy in 2005 to conserve and protect the environment. Pakistan is a member state in the Kyoto protocol agreement in which Pakistan vowed to control the CO_2_ emissions, to work on energy efficiency, and to tackle climate change problems in the country [6]. This study can provide possible outcomes of non-renewable energy projects in the future and can draw potential policy implications to support and add in the environmental laws of Pakistan.

Third, human development, technology, and population growth are novel addition in the existing literature. To our best knowledge, no empirical study has considered the nexus among selected variables for the case of Pakistan. By far, the studies conducted for Pakistan have overlooked mainly the human development aspect and concentrated primarily on the elements of economic growth and CO_2_ emissions. The present research determined to cover this research gap in the existing body of knowledge. The addition of human development, technology, and population in the model may result in a policy implication for Pakistan.

Finally, previous research used the ARDL methodology repeatedly to scrutinize the relation between total energy consumption, economic growth, and carbon emissions. This study incorporated novel variables and also employed several econometric techniques including fully modified ordinary least square (FMOLS) followed by Gregory and Hansen [22] structural break test to study the possible co-integration and robustness among variables. Additionally, the Granger causality approach under the VECM is employed to analyze the causal short and long-term relationships between the variables. This study explores the causal relationship between renewable and non-renewable energy consumption, environmental sustainability, economic growth, and the human development index (HDI) in Pakistan for the period 1990–2017. Thus, this study is equally important for academics and policymakers of emerging economies.

## 2. Literature Review

### 2.1. The Energy Consumption, GDP and CO_2_ Emission

The correlation between energy consumption, environmental degradation, and economic progress in a country is a crucial concern for researchers because fossil fuels contribute significantly to pollution [23]. Several econometric studies have analyzed the complex ties of economic activities with CO_2_ emissions and energy consumption [24,25,26]. Energy consumption is also a source for the intensifying the Earth’s temperature by overusing the fossil fuels emitting harmful gasses [27]. In Pakistan, a significant portion of energy is produced from non-renewable energy resources, causing a rise pollution, which may lead to environmental catastrophes [28]. Hassan et al. [29] investigated the significant drivers affecting long-run and short-run CO_2_ emissions on the bases of annual data from 1980–2011. The findings indicated a negative short-term relationship between economic growth and CO_2_ emissions. Naqvi and Rehm [11] focus on the casual association involving CO_2_ emissions, energy consumption, GDP, and population growth in Pakistan. The result revealed a long-run positive relationship among variables and also stated that a 1% increase in population, energy consumption, and GDP would increase emissions by 0.46%, 9.70%, and 0.005%, respectively.

Rafindadi [30] explores the linkage between economic growth and CO_2_ emissions for Nigeria. The study findings showed that financial growth increases energy demand but decreases CO_2_ emissions. Tiwari [31] used the Granger approach, and based on the findings, he argued that CO_2_ emissions are a byproduct of increasing economic activities and energy consumption in India. After studying the association between energy consumption and CO_2_ emissions, Apergis and Payne [4] discovered a significant correlation that CO_2_ emissions share with energy consumption in central America. Liu [32] investigated GDP’s impact on CO_2_ emissions and discovered a negative correlation of CO_2_ emissions with income. Niu et al. [33] studied eight Asian countries, and the results suggested a strong correlation is running from energy consumption to CO_2_ emissions. However, higher energy efficiency has been observed in developed countries compared to developing nations. Arouri et al. [34] researched 12 countries in the Middle East and reported a positive long-run relationship between energy consumption and CO_2_ emissions. Al-Mulali and Normee [35] targeted 19 countries and confirmed that a positive association exists between economic growth and energy consumption. The findings of Wei [36] revealed a long-run co-integration between energy consumption and economic variables. Total energy consumption is caused by economic growth in Pakistan [37]. Energy is a vital element for smooth economic activities and a critical source for production. Therefore, the consumption of energy and economic development are inextricably related [38,39,40]. Saidi and Hammami [26] studied the impact of CO_2_ and energy consumption on economic growth in 58 countries, and the results demonstrated that energy consumption is positively related to economic growth but increase CO_2_ emissions in longer run, which slows down the economic growth process.

Chien-Liang and Ting-Huan [41] found that renewables had a negative effect on CO_2_ emissions and also asserted to use more renewables to decrease CO_2_ emissions. Renewable energy has a substantial impact on economic and environmental sustainability, reduces reliance on non-renewable energy sources, and also serves as a tool for generating new job opportunities [42].

### 2.2. Energy Consumption, HDI, Population Growth, and Technology 

Higher energy consumption was earlier considered to be a tool to improve human development. According to Qiaosheng et al [43], the human development index (HDI) shares a direct association with energy consumption for developing countries. Steinberger and Roberts [44] argued that higher living standards are not merely a result of rising carbon emissions. HDI requires a moderate level of CO_2_ emissions to thrive, but an increase in HDI substantially associated with higher energy consumption. 

Serap and Vildan [45] researched 33 OECD countries to analyze the relationship between carbon emission and HDI. The results suggest that HDI is no affected by an increase or decrease in carbon emissions. However, the relationship between carbon emissions and human development varies from country to country. The findings of Martinez and Ebenhack [46] demonstrated that an increase in energy consumption could gradually increase the HDI in energy-poor countries. In contrast, energy consumption is moderately growing in transitioning countries. Energy affects the human development determinants (educational system, environmental protection, health, and gender equality) [47]. Economic growth plays a vital role in eradicating poverty and increasing per capita income, but it does not guarantee an improvement in the human development index [15,48].

Ang [49] and Qiaosheng et al. [43] showed that energy consumption per capita and indices for human development are closely linked in developing countries. According to Pirlogea [50], energy consumption is positively associated with and increases the human development index level, but it does not lead to sustaining higher HDI levels in the long run without meeting certain conditions. 

Thomas et al. [51] use the IPAT model to calculate CO_2_ emissions. They find that an increase in the population of 1% contributes to a rise in CO_2_ emission of 1%. Human beings are emitters of CO_2_; thus, population is directly linked to environmental degradation. If the living standards and the population in a country continue to increase, this will result in high energy consumption and CO_2_ emissions [52]. Garau et al. [53] explored the impacts of population on energy use and found that aged people in the population contributes to decreasing total energy usage. Energy demand is therefore determined exogenously by the population [26]. Shaari et al. [54] examined the association of population and energy consumption with economic growth and concluded that population and energy consumption share a significant relation, so population growth contributes to higher energy consumption. 

The development and implementation of technology in the production process raises energy consumption and has guided the industrialization and economic growth cycle [55]. Higher energy consumption is considered a critical factor in environmental challenges because higher consumption releases more elevated CO_2_ emissions into the atmosphere. The effect of emerging technology on energy consumption has important policy implications. Many proposals for environmental policy are expected to lead to the development of new technologies. The Clinton administration has made the development of more effective technologies one of the cornerstones of its proposal for the Kyoto climate change summit in 1997. Several plans for environmental policies, such as carbon taxes, also target energy consumption. Such a system increases the energy cost; more effort will be directed towards developing energy-efficient technologies [56,57]. The underlying characteristics of emerging technology positively affect energy and also help to smooth the road to environmental sustainability. However, setting proper goals and considering essential factors is necessary for beginning the path to sustainability [18]. A literature review reveals that non-renewable energy consumption is detrimental to the environment, human development, and long-term economic interests, but the results vary across countries. Based on a literature review, the present study addresses the causal relationship between renewable and non-renewable energy consumption, economic growth, environmental sustainability, and the human development index (HDI) in Pakistan for the period of 1990–2015. The study also forecasts the impact of energy projects under the umbrella of CPEC (China Pakistan economic corridor) using rigorous econometric techniques. The analysis incorporated population growth and technology as additional variables. Therefore, the present study addresses this crucial issue considering the combination of these factors for Pakistan in the short and long run.

## 3. Pakistan Energy Mix and Energy Projects Background in the Light of Belt and Road

**Figure 1:**
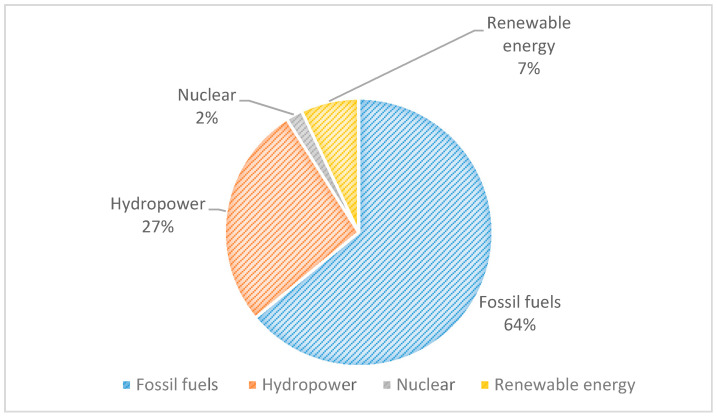
Pakistan Energy Statistics [63].

Pakistan is facing an acute energy crisis, which began in 2007, that has caused the country’s GDP to fall by 2% annually. Hundreds of factories (approximately 500) are either closed down in Faisalabad (the second largest industrial hub in Pakistan) or moved its operation in other countries (Bangladesh). Few Western companies have closed their operations in Pakistan. The unemployment rate has risen over the years and left millions unemployed across the country. Corruption, poor resource management, and lack of a strategic approach are a few factors that have worsened the situation [9,58]. Figure 1 indicates that Pakistan largely relies on traditional energy resources to meet its demands. The increased demand for fossil fuels in developing countries and its adverse environmental impact have forced policymakers and planners to consider environmentally sustainable alternatives. Over the past decade, a gradual change from non-renewables to renewables has been observed worldwide [59]. Renewable energy sources are emerging as intense competition for traditional sources of energy [60]. Unfortunately, in Pakistan’s current energy supply scenario, the renewable energy contribution constitutes just a small proportion of the overall percentage. Over the past decades, very little significant and remarkable effort has been made in Pakistan to utilize renewable energy resources. Pakistan has great potential for renewable energy utilization and appropriate infrastructure as that of Ireland or Denmark should be introduced to help the country in the best possible way [61,62].

Pakistan’s splendid geographical location in Asia has caught China’s attention to extend the relationship to a more interconnected association. In the past, Pakistan and China have collaborated on various projects, but in 2013, Chinese president Xi Jinping announced the world’s most significant project, the Belt and Road Initiative (BRI), in which Pakistan is one of the major stakeholders. China sought to develop six corridors under the BRI, and the China Pakistan Economic Corridor (CPEC) is one of its corridors, which will help connect Pakistan with the Western world. Under the umbrella of CPEC, China is investing billions of dollars for infrastructure (rail, road, and port) development in Pakistan [64].

**Figure 2:**
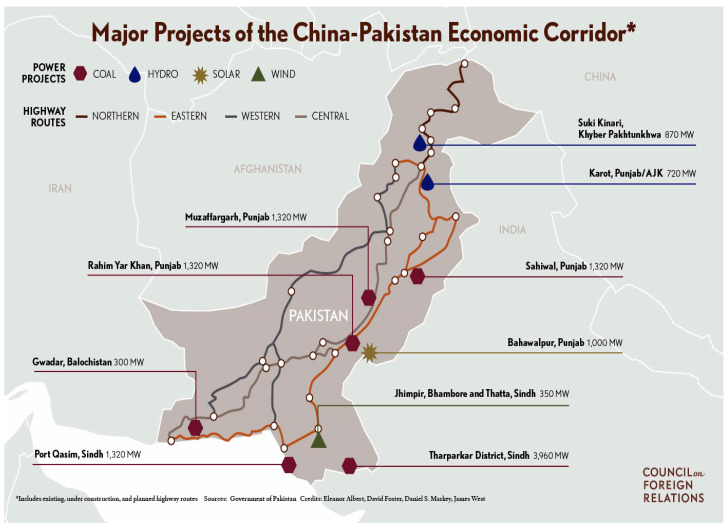
Energy projects under the China Pakistan Economic Corridor (CPEC) [21].

Figure 2 show that the CPEC is a mega project with a long-term investment ($64 billion USD) by China in Pakistan’s energy and infrastructure. Forty-six billion US dollars are allocated to develop 21 energy projects in Pakistan, in which 15 will be CPEC-priority energy projects, four will fall into the category of CPEC active energy promoted projects, and two will be potential energy projects. Pakistan needs a sufficient energy supply to alleviate the devastating economic conditions in Pakistan. Most of the projects under CPEC are non-renewables [65].

Pakistan’s economic growth depends on energy; despite renewable energy’s potential, Pakistan’s renewable plants cannot cover energy demands as much as non-renewables can. If energy demands are not fulfilled, the situation will worsen, as demands are increasing day by day and supplies are limited. Pakistan understands the dire need for energy sources and supplies for both social and economic development [13,14] Under CPEC, 67% of energy will be produced from non-renewable, which poses a significant threat to the environment. The world’s biggest economies have also utilized non-renewable energy for decades to reach where they are now. Non-renewable energy is still the most popular source of energy production globally. Coal is the largest carbon dioxide emitter (30% of all energy sources) [66,67]. In the current scenario and according to the statistics available, Pakistan follows that path, but renewable energy is the last resort. Therefore, based on the facts mentioned above, this study is focused on reinvestigating the energy, growth, environmental sustainability, and HDI for Pakistan.

## 4. Research Methodology

### 4.1. Data Source and Variables Description 

The main agenda of this article is to examine the relationship between renewable energy consumption (RE), non-renewable energy consumption (NRE), economic growth (GDP), environmental sustainability (CO_2_), human development index (HDI), technology (patent applications; PAs), and population growth (PG) for Pakistan. The present study has utilized time-series data from 1990 to 2017. The World Bank [68] and our world in data [69] and are used to extract data for study variables. Renewable and non-renewable energy consumption are calculated as a percentage of total energy consumption in kWh and values then converted into TWh. Economic growth calculated in the gross domestic product (GDP) (constant 2010 USD), and environmental sustainability estimated in the form of carbon dioxide emissions (mt of CO_2_). The variable of technology measured in the patent applications, denoted by PAs and population growth (PG) calculated in (% annual). The human development index (HDI) defined as a determinant of the core human development dimension: a stable life, good education, and a reasonable standard of living. It measured in terms of HDI for Pakistan and data collected from our world in data [69].

### 4.2. Unit Root Test

The Zivot and Andrews [70] approach is used for analyzing the stationary properties of data. The presence of a structural break in the data series leads to major forecasting errors. Perron [71] stated that conventional approaches could generate ambiguous conclusions about stationary structure in data. Zivot and Andrews [70] is a modified edition of the Perron [71] method, and this approach is unique as it addresses the systematic breaks on multiple data points. Therefore, it generates accurate estimates of the break series. The test equation is given as follows:

(1) $$∆X_{t}=bx_{t-1}+ct+bDT_{t}+\overset{k}{\underset{j=1}{\sum }}d_{j}∆X_{t-j}+\mu_{t}$$

(2) $$∆X_{t}=c+bx_{t-1}+ct+dDU_{t}+\overset{k}{\underset{j=1}{\sum }}d_{j}∆X_{t-j}+\mu_{t}$$

D$U_{t}$ denotes dummy variables used for a given point in the mean shift with time breaks, while D$T_{t}$ represents time breaks in the series. The null hypothesis for unit root break dates suggests that the series has a unit root with an unknown structural breakpoint. This test represents and tests all the possible breakpoints successively.

### 4.3. Fully Modified Ordinary Least Squares (FMOLS)

Philips and Hansen [72] developed the FMOLS test, and Pedroni [73] upgraded it. This approach is unique as it takes care of small sample sizes and allows researchers to remove biases in the data caused by serial correlation and endogeneity [74]. This technique is superior over ordinary regressions methodologies as it rectifies the inference problem and thus generates accurate long-run estimates. This test further helps to consider heterogeneity across segments. The designed models of the study are as follows:

(3) $$GDP_{t}=\beta_{0}+\beta_{1}RE_{t}+\beta_{2}NRE_{t}+\beta_{3}CO_{2}+\beta_{4}HDI+\beta_{5}PG+\beta_{6}PA$$

(4) $$CO_{2\;t}=\beta_{0}+\beta_{1}RE_{t}+\beta_{2}NRE_{t}+\beta_{3}GDP+\beta_{4}HDI+\beta_{5}PG+\beta_{6}PA\;$$

(5) $$HDI_{t}=\beta_{0}+\beta_{1}RE_{t}+\beta_{2}NRE_{t}+\beta_{3}GDP+\beta_{4}CO_{2}+\beta_{5}PG+\beta_{6}PA$$

In Equations (3)–(5), GDP, CO_2_, and HDI are dependent variables, whereas $E_{t}$ indicates an error term and $\beta_{0}$ refers to the intercept.

The FMOLS is an improved version of ordinary least squares (OLS), and it gives precise findings with more flexibility in several ways. The FMOLS incorporates the standard Wald test based on an asymptotic statistical analysis of Chi-square and considers both the endogeneity and serial correlation. This method offers investigators further options to identify the gaps among the two ways and provides an objective estimation of co-integrating regressions in a single equation. Moreover, this approach is asymptotically balanced and is ideal for mixed normal asymptotic behavior. The thorough discussions begin with a simple regression.

(6) $$Y_{t}=\beta_{0}+\beta_{T}X_{t}+\mu_{t}\;\;\;\;\;t=1\ldots n$$

The independent variables in Equation (6) are in order of I (1); further, the equations explain that variables are not cointegrated. In order to isolate the drift vector from a stationary variable, the stationary method for independent variables is required. The unit root and Gregory–Hansen test have already been applied to verify the co-integration. This study only used the FMOLS test when variables are co-integrated. The FMOLS method produces accurate estimates for small sample sizes and offers a robustness test for the findings. The FMOLS method used to estimate a single co-integrating relationship that has an I (1) combination.

### 4.4. Co-Integration Test

This study applies the Gregory–Hansen method to analyze the co-integration between variables. This approach uses a no co-integration hypothesis and is useful in case of a possible regime shift. If there is a break in intercept and slope coefficients, the Gregory–Hansen test can still identify the relationship between the variables of the study. The traditional augmented Dickey–Fuller (ADF) approach is not applicable or a good option for such conditions [22]. The three models in the Gregory–Hansen test incorporates numerous assumptions at the change in level or change in level with the trend and finally with the change in regime. The equations of the three models are as follows:

(7) $$Y_{t}=\mu_{2}+\mu_{2}f_{tk}+\beta_{1}t+\alpha_{1}X_{t}+\varepsilon_{t}$$

(8) $$Y_{t}=\mu_{1}+\mu_{2}f_{tk}+\beta_{1}t+\alpha_{1}X_{t}+\alpha_{2}X_{t}f_{tk}+\varepsilon_{t}$$

(9) $$Y_{t}=\mu_{1}+\mu_{2}f_{tk}+\beta_{1}t+b_{2}tf_{tk}+\alpha_{1}X_{t}+\alpha_{2}X_{t}f_{tk}+\varepsilon_{t}$$

This research used the Gregory–Hansen test to access the potential breaks and the dates of a break in the data. The highest absolute ADF test value determines break selection in the data. Then, the status of the series is examined by comparing the critical value with the calculated ADF test value. In the econometric model, X represents the independent variable, Y is dependent variables, and k is the break date in the data series. This study utilized all three models to check the impact of various factors on GDP, CO_2_ emissions, and HDI in Pakistan.

### 4.5. VECM and Causality Test 

The presence of a long-term relationship among the variables cannot provide details on cause and effect variables. For this purpose, the Engle and Granger [75] test is used to analyze the course of causality between the variables.

The Granger approach functions in such a way that if, in a time series data, δt is a possible cause of the other time series βt and if the δt’s previous values are contributing in forecasting βt, then we can conclude that δt is the granger causing βt. The direction of causality from βt to δt can also be explained in the same manner. The Vector Error Correction Model VECM model can determine the long and short-run relationship among the variables and this approach can further identify causative sources. The VECM model is given as follows:

(10) $$\begin{array}{l} {∆\mathrm{GDP}}_{t}\\ =\theta_{1i}+\overset{q}{\underset{j=1}{\sum }}\theta_{1,1\mathrm{ij},}{∆\mathrm{GDP}}_{\mathrm{it}-j}+\overset{q}{\underset{j=1}{\sum }}\theta_{1,2\mathrm{ij}}{∆\mathrm{RE}}_{\mathrm{it}-j}+\overset{q}{\underset{j=1}{\sum }}\theta_{1,3\mathrm{ij}}{∆\mathrm{NRE}}_{\mathrm{it}-j}+\overset{q}{\underset{j=1}{\sum }}\theta_{1,4\mathrm{ij}}{∆\mathrm{HDI}}_{\mathrm{it}-j}\\ +\overset{q}{\underset{j=1}{\sum }}\theta_{1,5\mathrm{ij}}{∆\mathrm{CO}}_{2}{\;}_{\mathrm{it}-j}+\overset{q}{\underset{j=1}{\sum }}\theta_{1,2\mathrm{ij}}{∆\mathrm{PG}}_{\mathrm{it}-j}+\overset{q}{\underset{j=1}{\sum }}\theta_{1,2\mathrm{ij}}{∆\mathrm{PA}}_{\mathrm{it}-j}+\lambda_{1i}{\mathrm{ECT}}_{\mathrm{it}-1}\\ +\mu_{\mathrm{it}} \end{array}$$

(11) $$\begin{array}{ll} {∆\mathrm{CO}}_{2}{}_{t}=\theta_{1i} & +\overset{q}{\underset{j=1}{\sum }}\theta_{1,1\mathrm{ij},}{∆\mathrm{GDP}}_{\mathrm{it}-j}+\overset{q}{\underset{j=1}{\sum }}\theta_{1,2\mathrm{ij}}{∆\mathrm{RE}}_{\mathrm{it}-j}+\overset{q}{\underset{j=1}{\sum }}\theta_{1,3\mathrm{ij}}{∆\mathrm{NRE}}_{\mathrm{it}-j}\\ & +\overset{q}{\underset{j=1}{\sum }}\theta_{1,4\mathrm{ij}}{∆\mathrm{HDI}}_{\mathrm{it}-j}+\overset{q}{\underset{j=1}{\sum }}\theta_{1,5\mathrm{ij}}{∆\mathrm{CO}}_{2}{}_{\mathrm{it}-j}+\overset{q}{\underset{j=1}{\sum }}\theta_{1,2\mathrm{ij}}{∆\mathrm{PG}}_{\mathrm{it}-j}\\ & +\overset{q}{\underset{j=1}{\sum }}\theta_{1,2\mathrm{ij}}{∆\mathrm{PA}}_{\mathrm{it}-j}+\lambda_{1i}{\mathrm{ECT}}_{\mathrm{it}-1}+\mu_{\mathrm{it}} \end{array}$$

(12) $$\begin{array}{ll} {∆\mathrm{HDI}}_{t}=\theta_{1i} & +\overset{q}{\underset{j=1}{\sum }}\theta_{1,1\mathrm{ij},}{∆\mathrm{GDP}}_{\mathrm{it}-j}+\overset{q}{\underset{j=1}{\sum }}\theta_{1,2\mathrm{ij}}{∆\mathrm{RE}}_{\mathrm{it}-j}+\overset{q}{\underset{j=1}{\sum }}\theta_{1,3\mathrm{ij}}{∆\mathrm{NRE}}_{\mathrm{it}-j}\\ & +\overset{q}{\underset{j=1}{\sum }}\theta_{1,4\mathrm{ij}}{∆\mathrm{HDI}}_{\mathrm{it}-j}+\overset{q}{\underset{j=1}{\sum }}\theta_{1,5\mathrm{ij}}{∆\mathrm{CO}}_{2}{}_{\mathrm{it}-j}+\overset{q}{\underset{j=1}{\sum }}\theta_{1,2\mathrm{ij}}{∆\mathrm{PG}}_{\mathrm{it}-j}\\ & +\overset{q}{\underset{j=1}{\sum }}\theta_{1,2\mathrm{ij}}{∆\mathrm{PA}}_{\mathrm{it}-j}+\lambda_{1i}{\mathrm{ECT}}_{\mathrm{it}-1}+\mu_{\mathrm{it}} \end{array}$$

(13) $$\begin{array}{ll} {∆\mathrm{RE}}_{t}=\theta_{1i} & +\overset{q}{\underset{j=1}{\sum }}\theta_{1,1\mathrm{ij},}{∆\mathrm{GDP}}_{\mathrm{it}-j}+\overset{q}{\underset{j=1}{\sum }}\theta_{1,2\mathrm{ij}}{∆\mathrm{RE}}_{\mathrm{it}-j}+\overset{q}{\underset{j=1}{\sum }}\theta_{1,3\mathrm{ij}}{∆\mathrm{NRE}}_{\mathrm{it}-j}\\ & +\overset{q}{\underset{j=1}{\sum }}\theta_{1,4\mathrm{ij}}{∆\mathrm{HDI}}_{\mathrm{it}-j}\;+\overset{q}{\underset{j=1}{\sum }}\theta_{1,5\mathrm{ij}}{∆\mathrm{CO}}_{2}{}_{\mathrm{it}-j}+\overset{q}{\underset{j=1}{\sum }}\theta_{1,2\mathrm{ij}}{∆\mathrm{PG}}_{\mathrm{it}-j}\\ & +\overset{q}{\underset{j=1}{\sum }}\theta_{1,2\mathrm{ij}}{∆\mathrm{PA}}_{\mathrm{it}-j}+\lambda_{1i}{\mathrm{ECT}}_{\mathrm{it}-1}+\mu_{\mathrm{it}} \end{array}$$

(14) $$\begin{array}{ll} {∆\mathrm{NRE}}_{t}=\theta_{1i} & +\overset{q}{\underset{j=1}{\sum }}\theta_{1,1\mathrm{ij},}{∆\mathrm{GDP}}_{\mathrm{it}-j}+\overset{q}{\underset{j=1}{\sum }}\theta_{1,2\mathrm{ij}}{∆\mathrm{RE}}_{\mathrm{it}-j}+\overset{q}{\underset{j=1}{\sum }}\theta_{1,3\mathrm{ij}}{∆\mathrm{NRE}}_{\mathrm{it}-j}\\ & +\overset{q}{\underset{j=1}{\sum }}\theta_{1,4\mathrm{ij}}{∆\mathrm{HDI}}_{\mathrm{it}-j}+\overset{q}{\underset{j=1}{\sum }}\theta_{1,5\mathrm{ij}}{∆\mathrm{CO}}_{2}{}_{\mathrm{it}-j}+\overset{q}{\underset{j=1}{\sum }}\theta_{1,2\mathrm{ij}}{∆\mathrm{PG}}_{\mathrm{it}-j}\\ & +\overset{q}{\underset{j=1}{\sum }}\theta_{1,2\mathrm{ij}}{∆\mathrm{PA}}_{\mathrm{it}-j}+\lambda_{1i}{\mathrm{ECT}}_{\mathrm{it}-1}+\mu_{\mathrm{it}} \end{array}$$

(15) $$\begin{array}{ll} {∆\mathrm{PG}}_{t}=\theta_{1i} & +\overset{q}{\underset{j=1}{\sum }}\theta_{1,1\mathrm{ij},}{∆\mathrm{GDP}}_{\mathrm{it}-j}+\overset{q}{\underset{j=1}{\sum }}\theta_{1,2\mathrm{ij}}{∆\mathrm{RE}}_{\mathrm{it}-j}+\overset{q}{\underset{j=1}{\sum }}\theta_{1,3\mathrm{ij}}{∆\mathrm{NRE}}_{\mathrm{it}-j}\\ & +\overset{q}{\underset{j=1}{\sum }}\theta_{1,4\mathrm{ij}}{∆\mathrm{HDI}}_{\mathrm{it}-j}+\overset{q}{\underset{j=1}{\sum }}\theta_{1,5\mathrm{ij}}{∆\mathrm{CO}}_{2}{}_{\mathrm{it}-j}+\overset{q}{\underset{j=1}{\sum }}\theta_{1,2\mathrm{ij}}{∆\mathrm{PG}}_{\mathrm{it}-j}\\ & +\overset{q}{\underset{j=1}{\sum }}\theta_{1,2\mathrm{ij}}{∆\mathrm{PA}}_{\mathrm{it}-j}+\lambda_{1i}{\mathrm{ECT}}_{\mathrm{it}-1}+\mu_{\mathrm{it}} \end{array}$$

(16) $$\begin{array}{ll} {∆\mathrm{PA}}_{t}=\theta_{1i} & +\overset{q}{\underset{j=1}{\sum }}\theta_{1,1\mathrm{ij},}{∆\mathrm{GDP}}_{\mathrm{it}-j}+\overset{q}{\underset{j=1}{\sum }}\theta_{1,2\mathrm{ij}}{∆\mathrm{RE}}_{\mathrm{it}-j}+\overset{q}{\underset{j=1}{\sum }}\theta_{1,3\mathrm{ij}}{∆\mathrm{NRE}}_{\mathrm{it}-j}\\ & +\overset{q}{\underset{j=1}{\sum }}\theta_{1,4\mathrm{ij}}{∆\mathrm{HDI}}_{\mathrm{it}-j}+\overset{q}{\underset{j=1}{\sum }}\theta_{1,5\mathrm{ij}}{∆\mathrm{CO}}_{2}{}_{\mathrm{it}-j}+\overset{q}{\underset{j=1}{\sum }}\theta_{1,2\mathrm{ij}}{∆\mathrm{PG}}_{\mathrm{it}-j}\\ & +\overset{q}{\underset{j=1}{\sum }}\theta_{1,2\mathrm{ij}}{∆\mathrm{PA}}_{\mathrm{it}-j}+\lambda_{1i}{\mathrm{ECT}}_{\mathrm{it}-1}+\mu_{\mathrm{it}} \end{array}$$

(17) $${\mathrm{ECT}}_{\mathrm{it}}=\beta_{1i}{\mathrm{GDP}}_{\mathrm{it}}+\beta_{2i}{\mathrm{CO}}^{2}{}_{\mathrm{it}}+\beta_{3i}{\mathrm{HDI}}_{\mathrm{it}}+\beta_{4i}{\mathrm{RE}}_{\mathrm{it}}+\beta_{5i}{\mathrm{NRE}}_{\mathrm{it}}+\beta_{6i}{\mathrm{PG}}_{\mathrm{it}}+\beta_{7i}{\mathrm{PA}}_{\mathrm{it}}$$

The lag length of auto-regression (q) is set at two, and the Schwarz information criterion (SIC) explains it automatically. ∆ is a first differential operator in the equation, µ defines random error term, and the ECT value represents the error correction term resulting from the long-run relationship. The Engle and Granger [75] approach is used to evaluate the complex relationship among variables for the short and long-term. F-statistics significantly determines the short and long-run causality, referring to the error correction term.

## 5. Empirical Results and Discussion

This study applied the Zivot–Andrews unit root test. This test is unique in considering structural breaks and includes details about its unexplained existence in the series. The test findings are presented in Table 1. The Zivot–Andrews test indicates that, in the presence of structural breaks, the variables comprise unit root problems. These breaks are 2013 for the series of renewable energy consumption and CO_2_ emissions; 2011 for the human development index and population growth; and 1999, 2002, and 2010 for non-renewable energy consumption, GDP and technology. The findings of the Zivot–Andrews test demonstrate the stationarity of the variables at the first difference.

### 5.1. Fully Modified Ordinary Least Square (FMOLS)

This study attempts to examine the long run estimation among the variables and thus apply FMOLS to estimate the connections among economic development, CO_2_ emissions, and HDI with the help of three separate models. The FMOLS findings are presented in the following tables.

In model one, GDP is considered a dependent variable, and the remaining variables are independent. The results in Table 2 showed both (renewable and non-renewable) energy resources share a significant relationship with GDP ensuring that both energy sources have a long-term impact on Pakistan’s economic growth. This result is similar to that of Shahbaz [12]. The significantly positive relationship of CO_2_ emissions with GDP precisely depicts that, in Pakistan, economic growth is affected by CO_2_ emissions. Pakistan has relied upon fossil fuels to meet most of its energy requirements. The negative coefficient value of non-renewable energy consumption implies that if Pakistan continues to depend on non-renewable for the longer run, it will cause an increase in CO_2_ emission and lower economic growth. These findings are similar to the results of several studies [5,14,76]. The amount of CO_2_ emissions can only be controlled by increasing renewable share in Pakistan’s overall energy mix. Legislators must emphasize the crucial part of renewable energy policy because renewables offer a solution to future energy demand and ensure long-run economic growth. Our model shows that population growth has no significant connection with economic growth in Pakistan. The human development index (HDI) and technology share a considerable relation with GDP in the longer run. Improvement in social conditions and well-being can trigger a boost in the economic growth of Pakistan. The positive coefficient of technology implies that more investment in energy efficient technologies would improve the financial actives in the long run.

Pakistan’s future dependence on non-renewable energy sources is evident from the China Pakistan economic corridor’s project, in which more energy will be produced from fossil fuels [65]. The above results prove that Pakistan must devise an energy mix, which gradually lowers its dependence on fossil fuels. Human development must be considered while developing economic growth plans for Pakistan, as it can serve as a tool to stabilize the long-run economy. Technology plays an important role, whether it is for renewable or non-renewable plants. Vast coal reserves are found in Tharparkar, Sindh province of Pakistan. These reserves are the primary input of one of Thar-coal-fired projects under CPEC, but this coal is loaded with sulfur dioxide, which can cause severe respiratory diseases [63,64,77]. Modern technology can help to remove the harmful substances from Thar coal and also serve as a tool for energy efficiency both in production and consumption of technology is employed in the initial stages of renewable energy development in a country. The economy might face the pressure of increase installation costs, but, in the long run, positive coefficient value ensures that renewable energy would increase the economic growth along with the improved environment in the long term [76,78].

In the second model, CO_2_ emissions are considered a dependent variable, while other variables are held as independent (Table 3). The more noticeable aspect of the result is a negative coefficient value of renewable energy in relation to CO_2_ emissions in Pakistan. This negative relational value imposes a policy implication for Pakistan. A rise in renewable energy share in Pakistan’s overall energy mix can reduce the amount of CO_2_ emissions and protect the environment from deterioration. A substantial negative technology to economic growth relationship emphasizes that technology innovation can help Pakistan in reducing CO_2_ emissions.

Today, escalating environmental pollution and lower economic growth levels are a byproduct of the overuse of non-renewables in Pakistan [14]. GDP and non-renewable energy consumption share a robust long-term link with CO_2_ emissions; this suggests that an increase in economic growth with the usage of non-renewable energy resources can cause a surge in CO_2_ emissions. Pakistan’s economy has relied on coal gas and petrol for an extended period, and a significant portion of the energy (64%) is produced from non-renewables; this is why economic growth over the years has worsened the environmental condition in the country. These results are similar to those of several studies [8,79,80].

However, the human development index does not share a considerable long-term correlation with CO_2_ emissions for the case of Pakistan, which depicts that better schooling, employment conditions, or lifestyle in the country do not affect CO_2_ emissions. The positive association among CO_2_ emissions and population growth is backed by the results of Asjad and Baig [81] and Khan et al. [82], which also found a positive correlation between population and CO_2_ emissions. Humans are natural CO_2_ emitters, and an increased population leads to more CO_2_ emissions.

The human development index is considered a dependent variable in the third model. The results in Table 4 show that renewable and non-renewable energy consumption and human development index do share a long-term association in Pakistan. The negative coefficient of non-renewable energy consumption implies that the usage of fossil fuels has negative impacts on the human development index. Thus, human well-being is influenced by energy resources in Pakistan. The contribution of renewables in Pakistan (Figure 1) is relatively small; therefore, it is not related to better HDI in Pakistan. The human development index in Pakistan is positively linked to the environment and GDP in Pakistan; these results are similar to the findings of Wang et al. [83]. A positive correlation between HDI and CO_2_ emissions is an indication that increased CO_2_ emissions will cause health conditions in Pakistan and thus impact the human development index. GDP coefficient values suggest that increase economic growth can improve the human development index; findings are the same as reported by Sinha and Sen [84]. By stabilizing economic growth, Pakistan can direct more plans for education and health and other social sectors. Previously results mentioned in Table 2 shows that increased consumption of fossil fuels can raise CO_2_ emissions, and this environmental condition has a direct impact on Pakistan’s HDI. Increasing the share of renewables is a way to protect the environment, and improved environmental quality contributes to better HDI. Population growth is negatively associated with HDI, so an increase in population may hinder the human development index in Pakistan. More than 50% of Pakistan’s population belongs to the middle class. It is not very promising, and an uncontrolled increase in population is a cause of poverty. It also restricts citizens’ access to better social facilities [85].

### 5.2. Robustness Check

This study checked the robustness of outcomes and investigated co-integration among renewable and non-renewable energy consumption, economic growth, CO_2_ emissions, and technology by employing the Gregory–Hansen [22] test. The outcomes indicate that in the presence of structural break, renewable energy and non-renewable energy consumption, human development index, CO_2_ emissions, and technology, and they depict the long-run cointegrated for Pakistan with GDP as a dependent variable. The variables are related in the long run in three scenarios, but the break dates vary. The results in Table 5 indicate that the ADF statistic is significant with the break date in 2007. The model with level shift and trend and model for regime also suggests that the ADF statistics of −6.13 and −7.48 are significant at a 5% level for the break date 1997 and 2011.

The second model used CO_2_ as the main variable and checked the association among variables. The findings are reported in Table 6. The association among these variables is still unchanged that supports long term connection. From Table 6, according to three statistics (ADF* and Z*), the presence of a co-integration relationship at the 1%, 5%, and 10 % significance level cannot be rejected, so the break is 2012 and 2011 is significant.

The results for the Gregory–Hansen co-integration test Table 7 indicate that when HDI is treated as endogenous, in the presence of structural break or regime shift, a co-integration relationship exists among variables for a long run over the sampled period in the case of Pakistan. The implications of the Gregory–Hansen results are that structural break should be taken into consideration when modeling long-run relationships of renewable and non-renewable energy consumption, GDP, CO_2_ emissions, HDI, population growth, and technology in Pakistan.

### 5.3. VECM and Granger Causality

If variables of a study share a long-term association, then granger causality tests can be applied irrespective of the order of integration order among the series [86]. In the final step of the analysis, this research employed Granger Causality, vector error correction model to catch the direction of the short run and long run causalities between renewable and non-renewable energy consumption, CO_2_ emissions, GDP and human development index for Pakistan. The causality findings are presented in Table 8.

The assessment from the VECM explains the existence of unidirectional causality among renewable energy consumption and CO_2_ emissions in the short and long run. From the Granger causality findings, it is noticeable that renewable energy consumption, CO_2_ emissions share a negative correlation. ECT’s negative value suggests that a long-run link exists among renewable energy consumption, CO_2_ emissions, and economic growth. The study findings indicate that increased use of renewable energy consumption should gradually decrease CO_2_ emissions, resulting in long-term clean and smooth economic growth for Pakistan; these results are the same as those in the studies of Latif et al. [9]. In the short run, both renewable energy and non-renewable energy consumption and GDP share at a significance level of 10% and 5%. Renewable energy consumption does not affect the human development index in the short run, but it is converging in the long run. When a country transits from a developing state to a developed country, more polices are designed to replace non-renewables with renewables, and the result will be a smooth economy that will work on improving human development in the country. At a significant level of 5%, non-renewable energy consumption is a granger cause of economic growth and CO_2_ emissions in the short run in Pakistan. Pakistan has been utilizing fossil fuels for decades, and lower renewable energy production capacity made Pakistan’s GDP rely more on non-renewables for energy consumption. Industrial sectors demand energy, and more energy production from fossil fuels increases CO_2_ emissions in both the short and long run, and CO_2_ emissions lower economic growth in the long term [5,13]. However, non-renewable energy consumption does not affect human development and does not share any significant relation with population growth and technology in the short run for Pakistan. Economic growth and non-renewable energy consumption share a bidirectional causality in the long run as Pakistan’s economy depends mostly on this traditional resource to fulfil energy requirements. 

**Figure 3:**
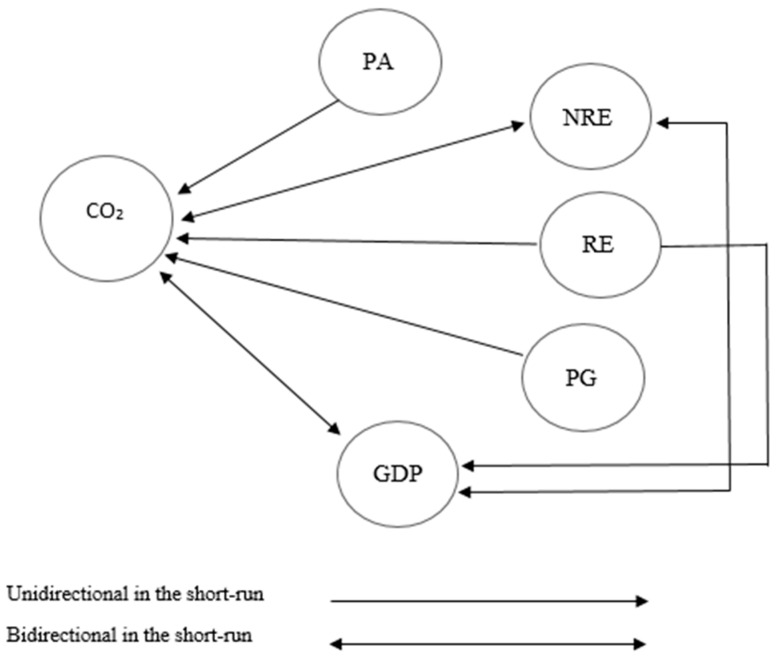
Short-run causality analysis.

**Figure 4:**
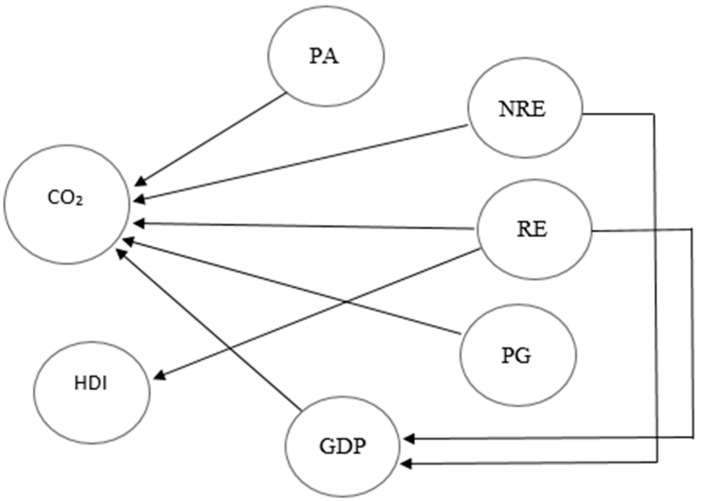
Long-run causality direction.

GDP is also a granger cause of CO_2_ emission in both short and in the long run (Figure 3 and Figure 4) because Pakistan’s economy depends on energy, which is mostly being produced from conventional energy resources. An increase in energy consumption will lead to higher coal, gas, and oil utilization to produce energy. Under the Pakistan China economic corridor, 67% of the power will be generated from non-renewables, which means that Pakistan has attached more priority of energy production with non-renewables [65,87].

In the short run, GDP does not exhibit a strong influence over renewable energy, and the relation between GDP and HDI is also insignificant in the short term. The human development index is not a granger cause of renewable energy, economic growth, and CO_2_ emissions in the short run. Population growth increases CO_2_ emissions in Pakistan in both the short and long term. The causality between population growth and CO_2_ emissions implies that an increase in population rate can cause a surge in CO_2_ emissions; however, CO_2_ does not cause population; these findings are similar to the results of Chandia et al. [80]. In the short run, technology and renewable energy do not share a causal relationship, but, in the long term, these two shares a bond in Pakistan, the empirical evidence of Geng and Qiang [88] also found the same. Technology shares a negative relation with CO_2_ emissions, which postulates that technology can cause a decrease in CO_2_ emissions for Pakistan.

## 6. Conclusions

Pakistan is striving to cover high energy demands to stabilize economic growth for the last couple of decades. Pakistan uses both renewable and non-renewable energy sources to produce energy; however, the non-renewable energy contribution (64%) in the overall energy mix is more massive than that of renewables (36%). The energy crisis, environmental issues, human development index, and slow pace of economic growth make Pakistan a suitable setting to study the causal relationship among these factors. The study has utilized annual data for the period 1990–2017 with a unit root, co-integration settings, and the Gregory–Hansen test to check the reliability of the FMOLS outcomes. The Granger causality with VECM specifications is also used to catch short-run and long-run causality among variables. The Zivot–Andrews test results indicate that structural breaks induce the unit root problems in the variables at level with various break years for each variable. However, the results at first confirmed the stationarity of the variables. Therefore, the unit root test fulfills the conditions for the next test, and the study uses FMOLS.

The results of FMOLS using GDP, HDI, and CO_2_ emissions as dependent variables indicate that non-renewable and renewable energy consumption shares a long-run relationship with Pakistan’s economic growth and CO_2_ emissions. Non-renewable energy is responsible for rising CO_2_ discharge; however, renewable energy consumption lowers CO_2_ emissions in the long run. HDI and technology show a positive relationship with economic growth showing that an increase in HDI will lead to a rise in GDP in Pakistan. The positive coefficient of technology with GDP and negative correlation with CO_2_ emissions implies that technology can also act as a catalyst to reduce CO_2_ emissions for Pakistan and helps to promote long-term economic growth. Humans are natural emitters of CO_2_ emissions, and the results confirmed that an increase in population both in the short and long run results in high CO_2_ emissions. Empirical results of the Gregory–Hansen test confirmed that variables are co-integrated and proved robust using GDP, CO_2_, and HDI as dependent variables. 

Finally, the current investigation finds that the bidirectional causality runs from non-renewable energy consumption, economic growth, and CO_2_ emissions in both the short and long run. Bidirectional causality also exits among renewable energy and economic growth. Furthermore, a unidirectional causality exists between renewable energy consumption and CO_2_ emissions in both the short and long run. HDI does not share short-run causality with any variable; however, it does converge in the long term with GDP, CO_2_ emissions, population growth, and technology. Thus, the causality outcomes are consistent with the FMOLS findings. This comparison suggests that results remain unchanged under various econometric approaches.

This study offers several policy implications regarding the nexus of these aforementioned variables. First, the long-run bidirectional causality among GDP and energy consumption from both sources (renewable and non-renewable) asserts that if Pakistan aims for a high degree of economic growth, sufficient energy supply is necessary. The government must make short, medium, and long-term plans to ensure adequate supply from respective energy sources in Pakistan. 

Second, Pakistan is facing an energy shortage, and the China Pakistan economic corridor (CPEC) is a solution to this energy crisis. However, energy projects under CPEC are mainly profit-driven and are not environment friendly. The government of Pakistan should develop and discuss environmental policies with China and convince the Chinese government to raise investment in cleaner technology.

Third, Pakistan must take economic and environmental prospects together. Traditional energy consumption will continue to increase in a developing country like Pakistan; however, technology can help to achieve sustainable development by slowly replacing non-renewable energy with renewable energy. Thus, policymakers must design the overall energy mix to take the best advantage of renewable potentials in Pakistan to achieve sustainable development.

Fourth, Pakistan needs to modify environmental laws strictly to withstand economic, environmental, and human development. The government must encourage industries to focus on environmentally friendly technology and make it a policy to grant loans to help enterprises to take steps that result in environment conservation.

Finally, this study reveals that the environmental conditions of Pakistan influence HDI. Therefore, before developing or imposing any energy project or policy, the establishment of Pakistan must take into account the long-term environmental, social, and economic interests of its citizens.

## Figures and Tables

**Table 1:** The Zivot–Andrews unit root test for structural breaks.

Variables	Test for Level		Test for First Difference	
	T-test	Break	T-test	Break
RE	−3.255	1994	−5.301 *	2013
NRE	−4.173	2008	−3.690	1999
GDP	−2.670 **	2002	−2.670	2002
CO_2_	−2.398	1996	−4.001	2013
HDI	−2.377	2011	−2.377	2011
Population growth (PG)	−6.174 *	2008	−6.754	2011
Technology (PA)	−4.000 *	2009	−3.986	2010

Note: * and ** is an indication of stationary behavior of variables at 5% and 10%, respectively.

**Table 2:** Fully modified least squares (FMOLS).

Dependent Variable: GDP				
Variables	Coefficient	Std. Error	t-statistic	Prob.
RE	2.180688	0.639943	1.844990	0.0007
NRE	−1.753553	0.507067	−3.458230	0.0025
CO_2_	0.838145	0.127810	6.557721	0.0000
HDI	39.2315	78.63266	4.822824	0.0001
PG	4.893752	7.202540	0.679448	0.5046
PA	25.46173	5.452700	4.669564	0.0001
C	−23.1247	67.60197	−1.821318	0.0836
R-squared	0.99746	Adjusted R-squared		0.996699

**Table 3:** Fully modified least squares (FMOLS).

Dependent Variable: CO_2_				
Variables	Coefficient	Std. Error	t-statistic	Prob.
RE	−1.237762	0.356984	−6.268521	0.0000
NRE	1.544231	0.359848	4.287174	0.0004
GDP	0.771803	0.094940	8.129376	0.0000
HDI	48.29915	68.07296	3.657715	0.0016
PG	1.044017	5.076727	−0.205648	0.0783
PA	−9.085110	5.664479	−1.603874	0.0544
C	−49.6310	45.24787	3.306918	0.035
R-Squared	0.994103	Adjusted R-Squared		0.992333

**Table 4:** Fully modified least squares (FMOLS).

Dependent Variable: HDI				
Variables	Coefficient	Std. Error	t-statistic	Prob.
RE	0.002227	0.001012	−2.200410	0.0397
NRE	−0.003353	0.000815	4.116154	0.0005
GDP	0.001366	0.000260	5.246001	0.0000
CO2	0.000981	0.000303	−3.231632	0.0042
PG	−0.022957	0.008968	−4.362057	0.0098
PA	0.22957	0.013015	−1.763943	0.0930
C	0.425098	0.085919	4.947687	0.0001
R-Squared	0.991174	Adjusted R-Squared		0.988526

**Table 5:** Gregory–Hansen test for co-integration with regime shifts (GDP): change in level.

Change in Level				
	Test Statistic	Breakpoint	Date	Critical Values
				1% 5% 10%
ADF	−7.05 ***	18	2007	−6.05 −5.56 −5.31
Zt	−7.46 ***	19	2008	−6.05 −5.56 −5.31
Za	−18.68	19	2008	−70.18 −59.40 −54.38
Change in Level & Trend				
ADF	−6.13 ***	8	1997	−6.05 −5.83 −5.59
Zt	−6.26 ***	15	2004	−6.05 −5.83 −5.59
Za	−31.71	15	2004	−76.95 −65.44 −60.12
Change in Regime				
ADF	−7.48 ***	22	2011	−6.36 −5.83 −5.83
Zt	−7.49 ***	24	2013	−6.36 −5.59 −5.59
Za	−37.59	24	2013	−76.95 −65.44 −60.12

Note: *** denotes the reject of the null hypothesis in 10%, 5% and 1%, respectively.

**Table 6:** Gregory–Hansen test for co-integration with regime shifts (CO_2_).

Change in Level				
	Test Statistic	Breakpoint	Date	Asymptotic Critical Values
				1% 5% 10%
ADF	−6.51 ***	23	2012	−5.77 −5.28 −5.02
Zt	−6.28 ***	24	2013	−5.77 −5.28 −5.02
Za	−24.09	24	2013	−63.64 −53.58 −48.65
Change in Level and Trend				
ADF	−7.29 ***	23	2012	−6.05 −5.57 −5.33
Zt	−6.54 ***	24	2013	−6.05 −5.57 −5.33
Za	−22.08	24	2013	−70.27 −59.76 −54.94
Change in Regime				
ADF	−7.52 ***	22	2011	−6.51 −6.00 −5.75
Zt	−7.39 ***	22	2011	−6.51 −6.00 −5.75
Za	−38.75	22	2011	−80.15 −68.94 −63.42

Note: *** denotes the reject of the null hypothesis in 10%, 5% and 1%, respectively.

**Table 7:** Gregory–Hansen test for co-integration with regime shifts (HDI).

Change in Level						
	Test Statistic	Breakpoint	Date	Critical Values		
				1%	5%	10%
ADF	−7.42 ***	23	2012	−6.05	−5.56	−5.31
Zt	−7.28 ***	24	2013	−6.05	−5.56	−5.31
Za	−24.09	24	2013	−70.18	−59.40	−54.38
Change in Level & Trend						
ADF	−8.23 ***	23	2012	−6.05	−5.83	−5.59
Zt	−7.99***	24	2013	−6.05	−5.83	−5.59
Za	−32.08	24	2013	−76.95	−65.44	−60.12
Change in Regime						
ADF	−7.52 **	22	2011	−6.36	−5.83	−5.83
Zt	−7.69 ***	22	2011	−6.36	−5.59	−5.59
Za	−38.75	22	2011	−76.95	−65.44	−60.12

Note: *** denotes the reject of the null hypothesis in 10%, 5% and 1%, respectively.

**Table 8:** VECM and Granger causality estimates.

Dependent Variable	Causation Source (Independent Variables)							
	Short run							Long Run
	∆RE	∆NRE	∆GDP	${∆\mathrm{CO}}_{2}$	∆HDI	∆PG	∆PA	
∆RE	–	0.0894	0.2610 *	−0.3382 **	0.0047	0.057	0.004	−0.753
∆NRE	0.423	–	0.169 **	2.2336 **	0.0019	0.021	−0.015	−0.293
∆GDP	0.164	0.039 **	–	0.6942 *	0.0222	0.002	0.047	−0.313
$∆{\mathrm{CO}}_{2}$	0.286	0.268 **	0.036 *	–	0.0631	0.089	0.092	−0.976
∆HDI	5.643	−13.80	9.568	44.0736	–	1.920	1.920	−1.248
∆PG	0.169	1.648	2.058	8.3734 **	0.5685	–	0.027	−8.415
∆PA	17.575	−1.887	8.748	−1.5399 **	0.0164	0.026	–	−1.559

*, **, *** denote the test significance at 10, 5%, and 1%, respectively.
